# Augmenting the anisotropic network model with torsional potentials improves PATH performance, enabling detailed comparison with experimental rate data

**DOI:** 10.1063/1.4976142

**Published:** 2017-02-16

**Authors:** Srinivas Niranj Chandrasekaran, Charles W. Carter

**Affiliations:** 1 Program in Bioinformatics and Integrative Biology, University of Massachusetts Medical School, Worcester, Massachusetts 01655, USA; 2 Department of Biochemistry and Biophysics, The University of North Carolina at Chapel Hill, Chapel Hill, North Carolina 27599-7260, USA

## Abstract

PATH algorithms for identifying conformational transition states provide computational parameters—time to the transition state, conformational free energy differences, and transition state activation energies—for comparison to experimental data and can be carried out sufficiently rapidly to use in the “high throughput” mode. These advantages are especially useful for interpreting results from combinatorial mutagenesis experiments. This report updates the previously published algorithm with enhancements that improve correlations between PATH convergence parameters derived from virtual variant structures generated by RosettaBackrub and previously published kinetic data for a complete, four-way combinatorial mutagenesis of a conformational switch in Tryptophanyl-tRNA synthetase.

## INTRODUCTION

Macromolecular crystal structures have been solved and deposited at an exponentially increasing rate ever since the Protein Data Bank (PDB) was established.^3^ But the number of novel structures that are being deposited has been decreasing.^26^ This suggests that increasing numbers of deposited structures are alternate configurations of macromolecules already in the database. The current situation lends itself to answering other interesting questions in structural biology, an important example being what are the intermediate structures that connect different equilibrium states that have been deposited to the PDB? Of these intermediate states, the most important is the conformational transition state structure, representing the ensemble of structures with the highest free energy along the conformational change pathway, and which can furnish information about the nature of barriers to conformational change processes, and by implication, their rates.

Computational approaches are especially relevant for enzymes where a significant body of experimental data exists implicating a large-scale and functionally important conformational change. *Geobacillus stearothermophilus* tryptophanyl-tRNA synthetase (TrpRS) is an example.^8^ It is useful to review two separate areas of our previous work, in order to understand the motivation for this revision of the PATH program. First, we summarize work on the enzymology of TrpRS and how its catalytic proficiency results from coupling to conformational changes.^7,24,27,39–42^ Work on the PATH program was undertaken because of the opportunity it presented to integrate these enzymological studies with computational analysis of the conformational transition state ensembles^9^ and thereby to connect structure to function both directly and indirectly via computational analysis.

### TrpRS enzymology

The accompanying paper^8^ describes measurement of three, distinct, long-range coupling energies—between enzyme and active-site Mg^2+^ ion, between the Mg^2+^ ion and four residues that undergo re-packing associated with domain movement, and between two auxiliary domains that move relative to one another during catalysis—that each contribute ∼−5 kcal/mol to transition-state stabilization during activation of tryptophan by *B. geostearothermophilus* TrpRS. The only consistent interpretation of these three coupling energies is that the active-site pre-organization and high transition-state affinity occur transiently during a large-scale conformational change.^8^

We began to study the reaction path using molecular dynamics to identify large scale domain motions.^22^ However, we could not identify any of the structural features defining conformational barriers. Subsequently, we rationalized those qualitative computational results by solving the crystal structure of an intermediate state in the induced-fit portion of the reaction path. That structure coincided with the structure predicted by Kapustina *et al*.^23^ and that identified as a transition state structure by earlier versions of the PATH program,^24^ providing the first suggestion that PATH might furnish significant new information about the reaction pathway.

### The PATH algorithm and the Onsager-Machlup action functional

The PATH algorithm^19^ rapidly finds the minimum of the Onsager Machlup (OM) action functional for the energy dissipation in an overdamped system, minimization of which allows identification of the most probable path taken by a dynamic system experiencing friction.^33^ We gained confidence with the PATH algorithm by showing that a trajectory computed over the entire path including both induced-fit and catalytic stages of the reaction using a version of the program designed to force a passage through an intermediate state (P. Koehl, personal communication) revealed that the volume surrounding the substrate tryptophan compressed in two stages imposing specificity for selecting the correct amino acid.^42^ The larger compression occurred near the transition state of the induced fit reaction and the second coincided equally closely with the transition state for the catalytic transition.^42^

Throughout these earlier studies, we had observed that at its stationary point the PATH action calculation estimated three parameters—the barrier height, the energy difference between initial and final states, and the time to the transition state—that might permit us to compare its accuracy quantitatively with experimental rate measurements for the 16 TrpRS variants developed in the combinatorial mutagenesis, paving the way to using computational models to assist in the identification and interpretation of structural barriers to conformational change. Several obstacles stood in the way of making quantitative use of the PATH trajectory parameters.

Some of these concerns were conceptual in nature. Others noted, as we had,^9^ that the systems spent inordinate amounts of the allotted time in the higher energy state, contradicting statistical mechanics.^18,20,30,35^ Skeptics^30,35^ also pointed out that the value of the Onsager-Machlup functional arises solely from the random forces acting on the system, thereby making all paths equally probable. This makes the action surface flat, especially near the conformational transition state, and therefore minimization of Onsager-Machlup action functional to identify the most probable path would not be possible. There also was discussion (J. Hermans, personal communication) of the problem posed by the fact that the conservation of momentum assumed in the transition state calculation was physically incompatible with the diffusive nature of the OM action functional.

We addressed some of these questions previously.^9^ In particular, (a) we used Discrete Molecular Dynamics^14,15,37^ with replica exchanges at different temperatures to map the free energy surface connecting the PreTS and Products states and showed thereby that the OM action minimization could indeed find a most probable path through an ensemble with a well-defined representative structure. (b) We showed that conformational changes for three distinct transitions—the TrpRS induced-fit transition, the myosin VI converter domain power stroke, and calmodulin calcium release—were all rate-limited by similar transition state ensembles in which aromatic residues needed to repack. (c) We showed that the transition states identified by PATH are the same as those generated by other specialized tools for studying this problem, such as the String method^16,34^ and ANMPathway.^11^

The most important problem with the previous PATH algorithm from our present standpoint, however, was that regression models using the PATH parameters gave poor correlations with experimental Δ*G_kcat_* values for the variant TrpRSs. A poor correlation with experiments meant that even though the dynamic trajectories generated using PATH agreed with other computational methods, its convergence parameters had no real world significance. We address this problem here, modifying the potential embedded in the Hessian matrix to represent torsional angle constraints and showing that results with the new Hessian matrix correlate well with experimental data.

The potential energy function that PATH uses is based on the Anisotropic Network Model (ANM),^1^ which has been successful in the study of protein conformational changes.^11,31,43^ However, as the “ball and spring” model cannot explicitly represent higher-order coupled atomic motions, it remains too simplistic to represent all relevant aspects of macromolecular conformational dynamics. Importantly, Na and Song^32^ showed that when augmented by potential energy terms to model geometrical and torsional constraints, the ANM potential performance approaches that of full potential MD force fields. Further, the conformational change trajectory in the previous implementation of PATH was computed as a function of time. As noted previously,^9,30,35^ when the trajectory is computed in this manner, it spends more time in the more energetic wells compared to the time it spends in the less energetic wells, which contradicts the laws of statistical mechanics. In Ref. 9, we suggested a heuristic approach where this problem could be avoided by initiating calculation of the trajectory only after the system begins to be displaced from equilibrium and ignoring the time the system spends near equilibrium. The problem with this approach is that the minimum displacement from the equilibrium position is defined arbitrarily and therefore the calculated trajectory is prone to errors. A more effective approach is to calculate the trajectory as a function of energy, instead of time.^17^

In this paper, we describe modifying PATH by augmenting the potential energy function to include additional interaction energy terms and revising the algorithm to calculate the trajectory as a function of energy, and removing the part of the trajectory that the system spends near equilibrium. We validate these algorithmic enhancements by using RosettaBackrub^25^ to generate a consistent set of virtual initial and final state structures for wild type and 15 combinatorial variants^8,41^ of TrpRS, as inputs to PATH and demonstrating high correlations between the PATH convergence parameters for this set, the pattern of mutant sites in each variant, and the corresponding experimental rate data. The comparison takes advantage of the rapid PATH algorithm, using it in the high-throughput mode to establish a potentially useful new window on protein conformational changes.

Construction of a torsional Hessian matrix and other modifications to PATH are described in the sections “Addition of a torsional Hessian to PATH” and “Calculating trajectories as a function of energy.”

## ALGORITHMIC MODIFICATIONS TO PATH

### Addition of a torsional Hessian to PATH

Macromolecules are typically modeled using complex potential energy functions that include several different types of interatomic interactions. The most common types of interactions found in most all atom force fields like AMBER,^10^ CHARMM,^5,6^ or Medusa^13,44^ represent bond stretching, bond bending, torsional, electrostatic, and Van der Waals potentials. Although these potentials give a complete description of macromolecules, the magnitude of the time steps at which the forces are integrated to calculate the velocities and atomic positions are in the order of femtoseconds. This limits the applicability of these energy functions to study the different kinds of dynamics that macromolecules undergo. This is true specifically in the case of low frequency large domain motions that are typical of macromolecular conformational changes.^1,2,11,38^

Coarse grained potential energy functions can partially solve this problem^28^ because they may use a subset of atoms, like only the C-alpha (CA) atoms to represent an entire amino acid^2,21^ thus increasing the speed of calculation. Further, protein conformational changes are often rigid-body motions of large domains relative to each other.^22^ Thus, a simplified potential like Elastic Network Model^38^ or ANM can be used to represent this motion. Also, because the integration timescale is appropriate for macromolecular conformational changes, ANM is well suited for studying this problem.

PATH^9^ currently uses an ANM potential to model interatomic interactions and to calculate the most probable conformational change pathway between two equilibrium states of a macromolecule. It uses a potential energy function^1^ that models atoms as hard, uncharged spheres and all interactions between atoms by vibrating springs, which are characterized by the same force constant, *k*. The ANM potential energy function is derived from Hooke's law and it is written as

$$V_{ANM}=\frac{k_{ANM}}{2}{\left(x-x_{0}\right)}^{2},$$ (1)

where *x*_0_ is the structure of the equilibrium state and (*x* – *x*_0_) is the displacement from the equilibrium state. This simple restoring force can approximate both bond length and bond angle constraints but cannot distinguish different kinds of interactions by modeling coupled motions between atoms in macromolecules.

With the ANM potential, PATH trajectories qualitatively explain experimental evidence that amino acid specificity is imposed in the transition state by showing that the volume surrounding the TrpRS amino acid substrate assumes a minimum in that state.^42^ However, the all-atom ANM potential does not accurately describe differences between sidechain and backbone motions, especially their frequencies. Also since ANM considers all interatomic interactions to be similar, it cannot provide a complete description of the motion of atoms in sidechains that may be involved in the conformational barrier. As shown by Na and Song,^32^ a trajectory from an all atom ANM model has a correlation of only about 46% to a trajectory generated from a full MD-like potential Normal Mode Analysis (NMA).

Na and Song show that the correlation between trajectories generated using ANM and full potential NMA increases rapidly to 79% once the torsional component is added to the potential energy function. Improvement is minimal for other additional terms until force field dependent components are added to the potential energy function, which eventually increases the correlation to 88% for sbNMA. Since the torsional terms account for the biggest improvement, are computationally inexpensive, and do not compromise the speed of PATH calculations, we decided to incorporate the torsional component into the ANM potential.

To include the torsional potential to the potential energy function of PATH, the potential has to be expressed in the form of a Hessian matrix. Construction of that hessian matrix is described in Appendix A.

### Introducing approximations to CA only simulations

In the case of CA only PATH simulations, often performed for very large systems to improve the speed of computation, the potential can be improved by modeling the potential energy function based on the dynamics of a macromolecule in a full MD potential. Currently, the ANM potential used for CA atoms assumes that a quadratic function models the energy surface of a macromolecular structure that is undergoing conformational change. It also assumes that the strength of interactions between any pair of atoms decays exponentially with the distance of separation between them. But as described in Ref. 21, when the dynamics of C-phycocyanin was studied using AMBER94^10^ and the CA pair distance was plotted, it was observed that the distances could be modeled in two separate regimes and the force constant for interaction in these two regimes can be calculated using the following empirical equations

$$k\left(r\right)=\{\begin{array}{cc} 8.6\times 10^{5}\;\text{kJ}\;{\text{mol}}^{–1}\;{\text{nm}}^{-3}\times r-2.39\times 10^{5}\;\text{kJ}\;{\text{mol}}^{–1\;}{\text{nm}}^{-2} & \text{for}\;r<0.4\;\text{nm}\\ 128\;\text{kJ}\;{\text{nm}}^{4\;}{\text{mol}}^{–1\;}r^{-6} & \text{for}\;r\geq 0.4\;\text{nm}. \end{array}$$ (2)

The Hessian matrix can then be built by incorporating these force constants into the ANM potential and weighting them by the mass of the two atoms in an atom pair. We call this Hessian matrix AMBER-based Mass Weighted Empirical Hessian (AMWEH)

### Calculating trajectories as a function of energy

PATH calculates trajectories between two equilibrium states of a macromolecule as a function of time. This makes PATH unique when compared with other similar methods like Plastic Network Model^31^ or ANMPathway,^11^ which do not furnish any time-related information and therefore cannot be used to estimate the (relative) rate of a conformational change reaction. The trajectory equation^9^ in PATH can be written as

$$x\left(t\right)=a+\frac{1}{\sinh\left(\Gamma \left(t_{2}-t_{1}\right)\right)}\left(\left(x_{1}-a\right)\sinh\left(t_{2}-t\right)-\left(x_{2}-a\right)\sinh\left(\Gamma \left(t_{1}-t\right)\right)\right),$$ (3)

where Γ is the force constant, *k* (for a one dimensional system) or the eigenvalue of the Hessian (in higher dimensions), *x*_1_ and *x*_2_ are the states the system is in at times *t*_1_ and *t*_2_, respectively, and *a* is the equilibrium state.

**FIG. 1.**
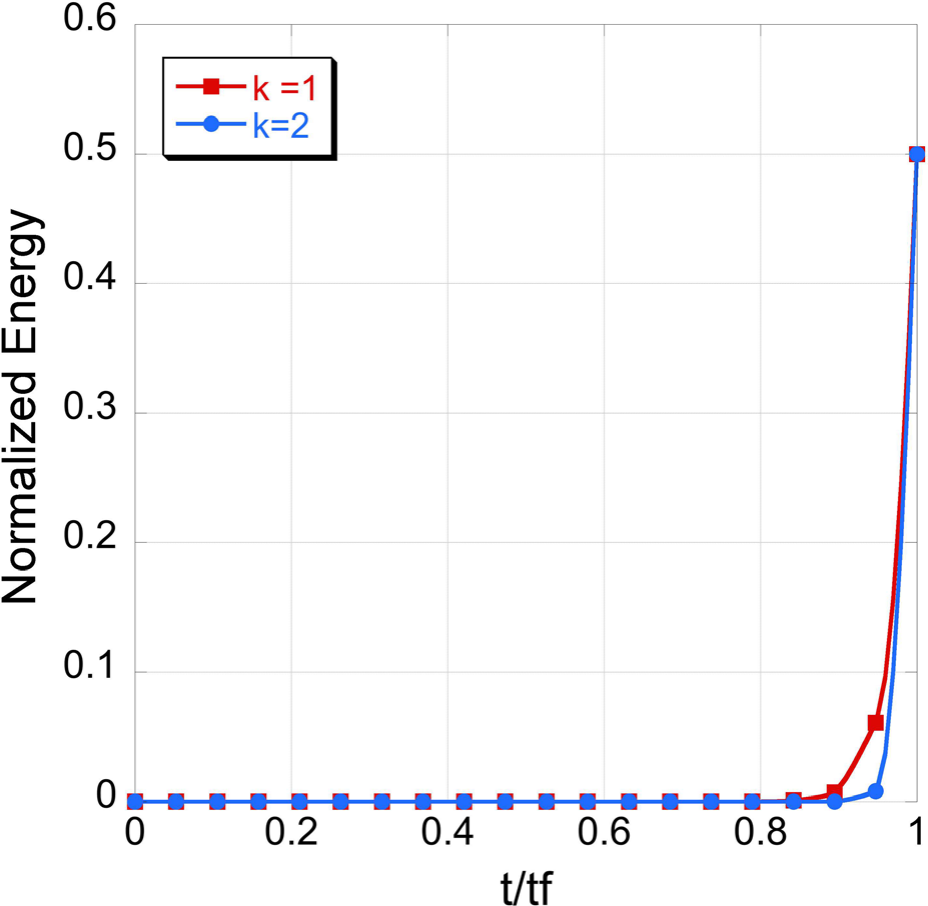
Simulations were performed with a diatomic system in the 1D heuristic model described in Ref. 9. Coordinates of the two end states are (Atom 1=(1,0,0), Atom 2=(−1,0,0)) and (Atom 1=(0.5,0,0), Atom 2=(−0.5,0,0)). The two simulations were identical except for the force constants (*k* = 1 and *k* = 2). The system with the smaller force constant takes longer to reach the end state once it is displaced from the equilibrium state (indicated by an increase in the energy) compared to the system with the larger force constant. The energy of the states in the trajectories is normalized relative to the force constants.

PATH requires that the system be provided with enough time to converge, so that the structure of the transition state becomes invariant for any additional time provided to the system.^9^ Since PATH assumes two potential energy wells, one for each equilibrium state, there are two trajectories *x_l_*(*t*) and *x_r_*(*t*) that intersect at the transition state. When the trajectory is calculated separately in the two wells using Equation (3), the system spends most of its time near the equilibrium states, taking only a small amount of time to climb the energy barrier to reach the transition state. Moreover, the system spends more time at equilibrium when the well is narrower (more energetic) compared to when the well is wider (Fig. 1).

This is contradictory to the behavior predicted by statistical mechanics, according to which, a system spends less time in the more energetic (larger force constant) well compared to a less energetic (smaller force constant) well. This impedes the ability of PATH to estimate relative reaction rates correctly.

**FIG. 2.**
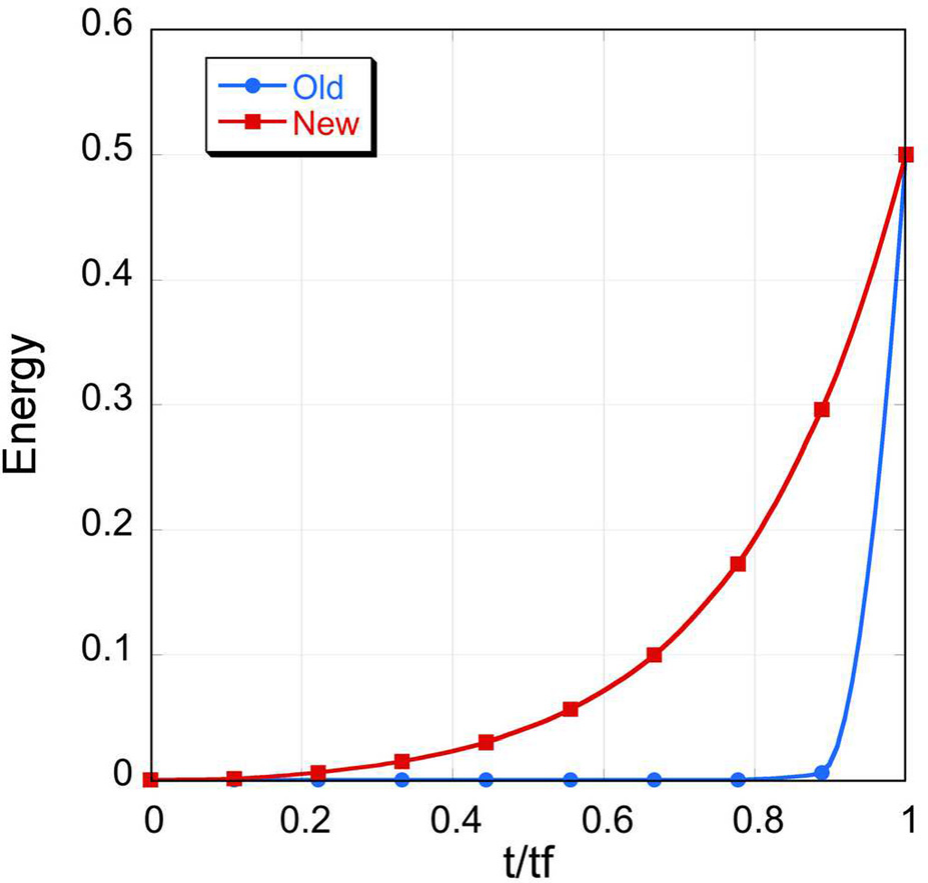
The same model described in Fig. 1 was used for these simulations. The structures in the trajectory generated with the new algorithm^9^ are different from the end states while the one generated with the old algorithm^19^ resembles the end states with sharp transitions between states.

PATH's disagreement with statistical mechanics is consistent with previous observations of groups working with the Onsager-Machlup action functional^35^ or other similar concepts.^18,20^ This was also one of the primary reasons for Ref. 30 to suggest that calculation of most probable paths by minimizing the Onsager-Machlup action functional may not generate meaningful pathways. We believe that this disagreement with statistical mechanics occurs primarily because the system has been provided with more time than it requires to converge. In Ref. 9, we suggested that the time the system spends near equilibrium could be removed from the trajectory such that the trajectory begins only after the system begins its ascent towards the transition state (Fig. 2). We based this approach on the observation (Fig. 1) that once the system begins to move away from equilibrium, it spends less time reaching the transition state from the equilibrium state in the more energetic well, which restored consistency with statistical thermodynamics. This heuristic approach removed any excess time that is provided to the system.

However, to accomplish this, we assumed, arbitrarily, that the trajectory began only after the system was displaced from equilibrium by at least 10% of the total distance between the equilibrium state and the transition state. Then, we derived the equation for the time to the transition state in Ref. 9 as

$$\bar{t}=\frac{2.303}{\bar{k}},$$ (4)

where $\bar{k}$ is the average force constant of the system, which is calculated from the mean of the eigenvalues of the Hessian matrix.

The arbitrary nature of Equation (4) made it a less convincing solution especially since other more complete solutions exist,^17^ where the time dependent Lagrangian is transformed to an energy dependent Hamilton-Jacobi description. Inspired by this approach, we transformed the equation to calculate the time to the transition state, $\bar{t}$, into the energy domain, which resulted in the following equation:

$$t=\frac{1}{\bar{k}}\left[X+\frac{1}{2}\ln\left(\frac{U\left(x\right)}{U\left(\bar{x}\right)}\right)\right],$$ (5)

where *U*(*x*) is the energy of the state *x* at time *t* and $U\left(\bar{x}\right)$ is the energy of the transition state. The derivation of this equation can be found in Appendix B.

As *U*(*x*) ≠ 0 only when the system moves away from equilibrium, this equation ensures that the time, *t*, is counted only when the structure is displaced from equilibrium and it works for all values of *X* (Fig. 2).

### Calculating mean force constants

Calculation of the mean value of force constants is crucial for calculating the conformational change pathway with realistic and PATH parameters. In Ref. 9, we calculated the mean force constant as the mean of the eigenvalues of the Hessian, which is also the mean of the trace of the Hessian matrix. This mean force constant is henceforth called *Trace*.

A drawback of this approach is that the eigenvalues that contribute most to the mean are the larger eigenvalues (high frequency modes). This results in a mean force constant that is far from the eigenvalues of modes that actually contribute to the overall conformational dynamics of the protein.

One way to increase the contribution of the smaller (low frequency modes) to the mean force constant is to use the largest vibrational mode (First Principal Component or *FPC*) to represent the force constant of the equilibrium state. Although this is a better representation of the mean force constant, often the first principal component can account for only a quarter of the total variation in the structure along the trajectory. Therefore, there are additional modes whose values have to be included while calculating the mean force constant.

A better approximation to the mean force constant representing all interactions in a system is a combination of the Trace and FPC methods, which we call *Inverse mean* (*IM*) method. Rather than calculating the mean of the trace of the Hessian, we calculate the inverse of the mean of the eigenvalues of the inverted Hessian matrix, which is the covariance matrix. Since low frequency modes have larger eigenvalues when they are calculated from the covariance matrix, this approach guarantees that the mean force constant has a higher contribution from low frequency modes. Also, to eliminate contributions from high frequency modes, only the largest (low frequency) modes that account for 95% variation in the structures along a trajectory are used to calculate the mean. Indeed, we find that 86% of the modes are necessary to account for 95% of the structural variation.

## Validation of PATH

### Calculating experimentally measurable parameters using PATH

Results from PATH were previously validated^19^ by demonstrating that the distance between CA atoms in the PATH trajectory remained within the range of experimentally observed values. We provided more convincing validation^9^ by comparing the PATH results with other computational algorithms like ANMPathway, using Replica Exchange Discrete Molecular Dynamics simulations to map the free energy surfaces linking the two ground states, and showing that PATH identified the transition state close to the saddle point of that surface.

From our earliest studies of the PATH algorithm, we have been tantalized by the possibility of validating it definitively, by correlating PATH-derived parameters with experimental data. Dellago *et al*.^12^ and Bolhuis *et al*.^4^ provide an in depth description of this problem, using ideas related to those outlined in our earlier paper.^9^ They, however, sought to compute rate constants directly from their simulations. Our objective is more modest: to validate the PATH results by demonstrating correlations of the PATH convergence parameters and experimental activation free energies. Thus, we do not seek absolute rate calculations but use rate measurements from an extensive set of related variants to establish comparable relative rates. To secure such validation, we considered values from PATH simulations that can be related to experimentally measured data. There are four such parameters, three of which—the time to the transition state, the energetic difference between initial and final state, and the barrier height—are provided directly by the PATH algorithm.

The fourth such value is the Gibbs free energy change associated with the conformational change itself, whose derivation and relevance to kinetic processes are both less obvious. We calculate the free energy difference between the two end states of a macromolecule by calculating the rate of reaction using the Arrhenius equation

$$r=Ae^{-\frac{\Delta U}{k_{B}T}},$$ (6)

where Δ*U* is the difference in potential energy between an equilibrium state and the transition state and *A* is the collision frequency.

For a diatomic system, this collision frequency is the frequency of vibration of the interaction between the two atoms, given by the force constant or the eigenvalue of the Hessian matrix. Based on this assumption, for the whole macromolecule, we can relate *A* to the mean force constant of the molecule, $\bar{k}$, as

$$A=C\bar{k}.$$ (7)

Therefore, the Arrhenius equation is rewritten as

$$r=C\bar{k}e^{-\frac{\Delta U}{k_{B}T}}.$$ (8)

This equation has the same form as the equation for mean first passage time derived in Refs. 29 and 45, according to which the constant *C* is proportional to the ratio of the determinants of the Hessian matrices of the transition state and the equilibrium state. In our studies of the Hessian matrices with the ANM potential, we have always found determinants of the Hessian matrices to be almost identical for similar structures and that the ratio does not deviate far from unity. Hence for the purpose of calculating the rate of the reaction, we assume *C* to be the same for both the forward and the reverse reactions.

Then, the rates of the forward reaction (*l*) and the reverse reaction (*r*) are then written as

$$r_{l}=C{\bar{k}}_{l}e^{-\frac{U_{l}^{‡}}{k_{B}T}},$$ (9)

$$r_{r}=C{\bar{k}}_{r}e^{-\frac{U_{r}^{‡}}{k_{B}T}}.$$ (10)

Since the conformational free energy is a function of the equilibrium constant, *K_eq_*, it can be written as

$$\Delta G_{conf}=-k_{B}T\;\ln\left(K_{eq}\right),$$ (11)

where $K_{eq}=\left(\frac{r_{l}}{r_{r}}\right)$. Substituting Equations (9) and (10) into (11), we get

$$\Delta G_{conf}=-k_{B}T\ln\left(\frac{{\bar{k}}_{l}}{{\bar{k}}_{r}}\right)+k_{B}T\frac{U_{l}^{‡}-U_{r}^{‡}}{k_{B}T}.$$ (12)

As $U_{r}^{‡}-U_{l}^{‡}=\Delta E$

$$\Delta G_{conf}=-k_{B}T\ln\left(\frac{{\bar{k}}_{l}}{{\bar{k}}_{r}}\right)-\Delta E.$$ (13)

Or because of the relationship between $\bar{k}$ and $\bar{t}$ from Equation (4),

$$\Delta G_{conf}=-\Delta E-k_{B}T\;\ln\left(\frac{{\bar{t}}_{r}}{{\bar{t}}_{l}}\right).$$ (14)

Ideally, the conformational free energy in Equation (14) and how it changes upon mutation should, in principle, be measurable experimentally. However, because of the difficulty of resolving ligand-binding and conformational effects we have been able only to estimate this value indirectly, from differences in the affinity of different nucleotide-phosphate ligands.^36^ That procedure gave an estimate of ∼−3 kcal/mol for the PreTS to Product conformational change. Such approaches are impractical in the high-throughput mode required to process all 16 TrpRS variants.

The regression analyses discussed below imply that estimates for Δ*G_conf_* are well correlated with the presence of mutants in the combinatorial series (Fig. 4) and that the overall free energy change of the conformational change does indeed influence the experimental rate in combination with the other computationally derived parameters (Fig. 5, Table II). Due to the various approximations made earlier and the simplification of the potential energy function of PATH, a good correlation with experimental results can be expected only when other PATH parameters are included in the comparison with experiments, as developed in “Comparison with experimental results.”

### Comparison with experimental results

The parameters that characterize stationary behavior of the PATH algorithm (the mean transition state barrier-height, $\left\langle U^{‡}\right\rangle$; the difference between the forward and reverse barrier heights, Δ*E*; the overall free energy change of the conformational transition, Δ*G_conf_*; and the times to reach the transition state from the reactants and from the products states, (${\bar{t}}_{l},\;{\bar{t}}_{r}$) all potentially have physical significance. In the accompanying paper,^8^ we describe what appears to be a uniquely suited dataset for this purpose: 16 TrpRS variants involving full combinatorial analysis of four residues in a broadly conserved molecular switching region—the D1 Switch—in which the shear of TrpRS domain movement leads to side-chain repacking. Experimental steady-state and single-turnover kinetic analyses of the four-way factorial design of this set of mutant proteins furnish an exacting test of correlations because we previously established that the four mutated D1 switch residues from this motif compose the conformational transition state for the induced-fit portion of the structural reaction cycle.^9^

**FIG. 3.**
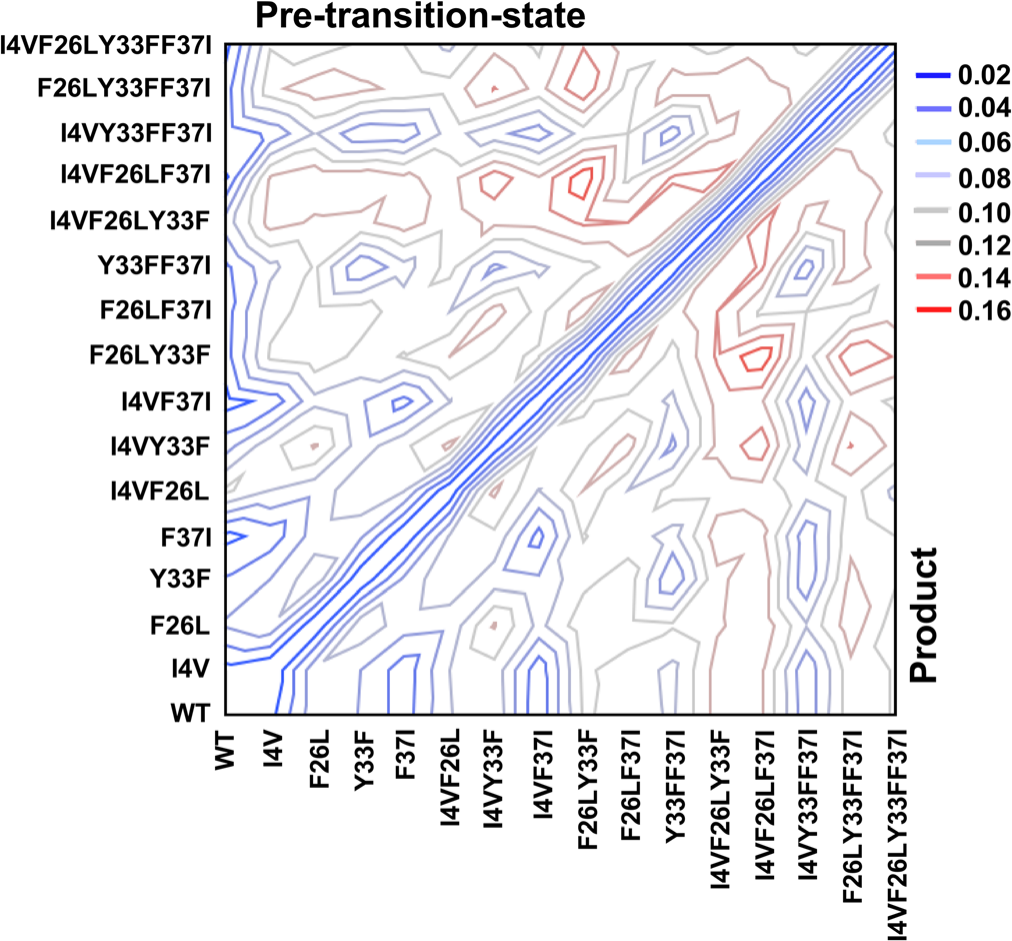
Root mean square differences between virtual mutant coordinates and those of the WT Pre-transition state (upper triangle) and Product (lower triangle) crystal structures.

Evaluating PATH convergence parameters for these variants required their atomic coordinates. Crystal structures have been solved in the PreTS state for six variants, including the quadruple mutant (T. Williams, personal communication). These structures suggest that the mutations induce minimal structural changes (root-mean-squared deviations (RMSDs) < 0.3 Å). Reasoning that the important requirement was that all coordinate sets satisfy the same potential functions, we generated atomic virtual coordinate sets for all 16 variants in the PreTS and Products forms. Virtual mutations were thus made to the crystallographic coordinates for the PreTS and Products states (PDB IDs 1MAU and 1I6 K) using the RosettaBackrub algorithm,^25^ which mutates a given amino acid and, in order to accommodate the new amino acid, alters the backbone of the protein minimally in the neighborhood of the mutation site. The program generated 20 structures and ranked them based on a scoring system, from which we chose the structures with the best scores. In order to generate a wild type structure conforming to the same potential functions, we reverted the virtual I4V mutant structure to wild type using the same program. The computationally designed mutant structures differ from the wild type structure at the mutant sites with an average RMSD of about 0.1 Å (Fig. 3). Further, although deviations are slightly larger for the product state structures, the symmetry across the diagonal in Fig. 3 implies that the mutations make comparable perturbations to the structures of both states. These RMSDs indicated that the virtual mutant structures are consistently related to the individual mutational perturbation at the different sites in the initial and final states. The all-by-all table of RMSD values provides 256 values and hence is a rich source of information about mutationally induced structural perturbation, which we have analyzed only to the extent that the largest contributors to the RMSD are the presence of F26L and Y33F mutations to both states, which together with their two-way interactions account for 62% of the variation in RMSD with Student t-test probabilities ≪10^−6^. The RMSDs and the structures are available in pymol session format as supplementary material.^46^

**FIG. 4.**
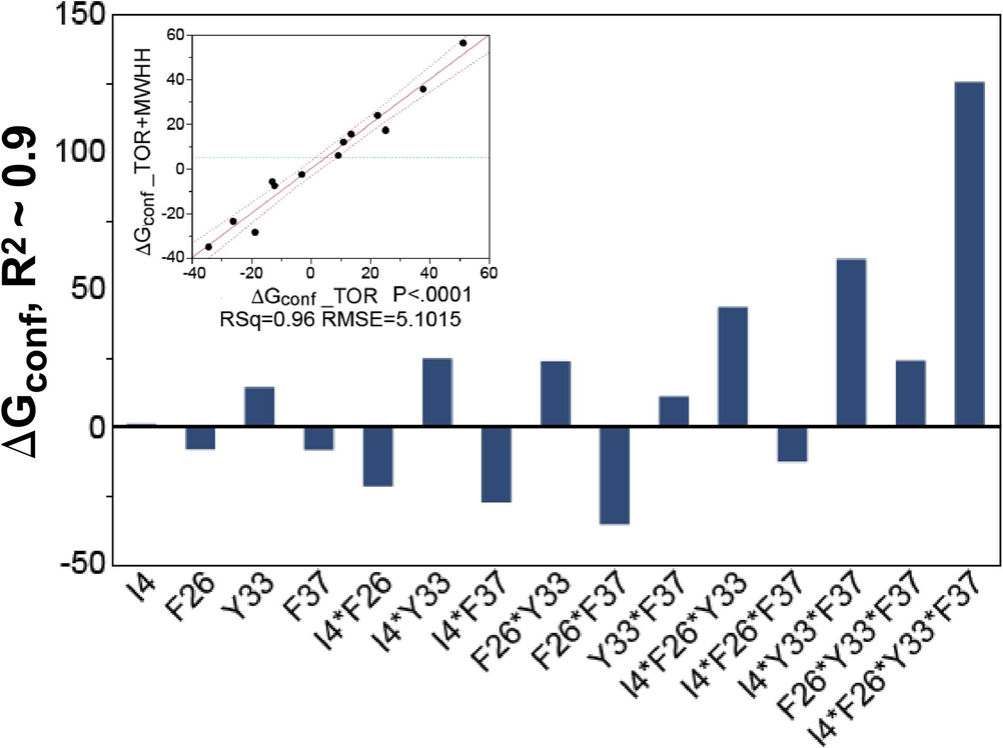
PATH estimates of Δ*G_conf_* (14) are closely correlated with the structural differences between virtual variants. PATH estimates of Δ*G_conf_* were obtained from the TOR_IM and AMWEH_IM algorithms and scaled by regression methods to account for differences between the all-atom TOR_IM (all atom) and AMWEH_IM (CA only) Hessians. Their units and those on the Y axis here are in arbitrary units. The histogram shows coefficients (in the same arbitrary units) for the regression model relating these Δ*G_conf_* values to the presence or absence of mutated sites in the virtual mutants. The most important contributor is the four-way interaction between I4 and the three aromatic side chains. The sign of this interaction indicates that, together, they make the overall Δ*G_conf_* less favorable, which is consistent with the experimental estimate and with the fact that the mutations were selected by the Rosetta multistate algorithm to reduce the free energy change between PreTS and Product states. The inset shows that the histogram is virtually identical to that obtained by fitting the TOR_IM derived Δ*G_conf_* values alone, for which the equal number of data and parameters precludes estimation of errors.

**FIG. 5.**
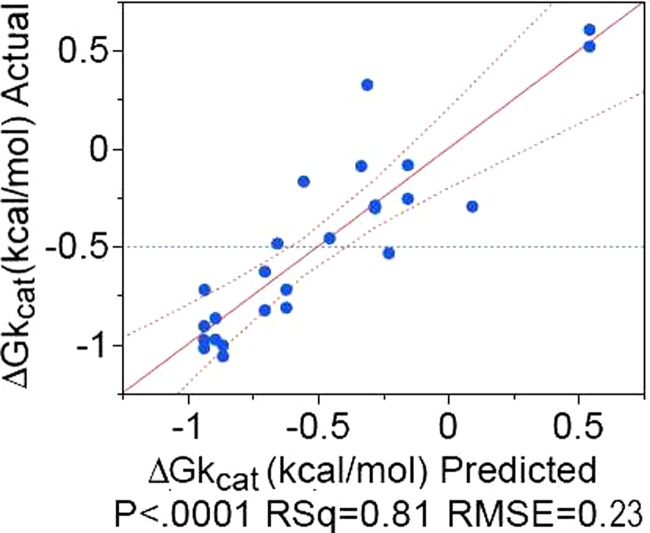
Regression model predicting experimental Δ*G_kcat_* values using three PATH convergence parameters. Experimental data are plotted against values predicted using coefficients in Table I.

These coordinate sets were then input to the PATH algorithm to generate parameter sets corresponding to each virtual variant. We show now that the PATH convergence parameters are consistently correlated both with the mutated TrpRS sites (Fig. 4) and with the experimental Δ*G_kcat_* values of the mutant proteins (Fig. 5).

To help ensure that PATH consistently reflects differences between the virtual structures, we investigated the correlation between overall free energy difference, Δ*G_conf_*, estimated from PATH using (14) and the presence of native or mutant side chains at the four mutated sites (Fig. 4).

**FIG. 6.**
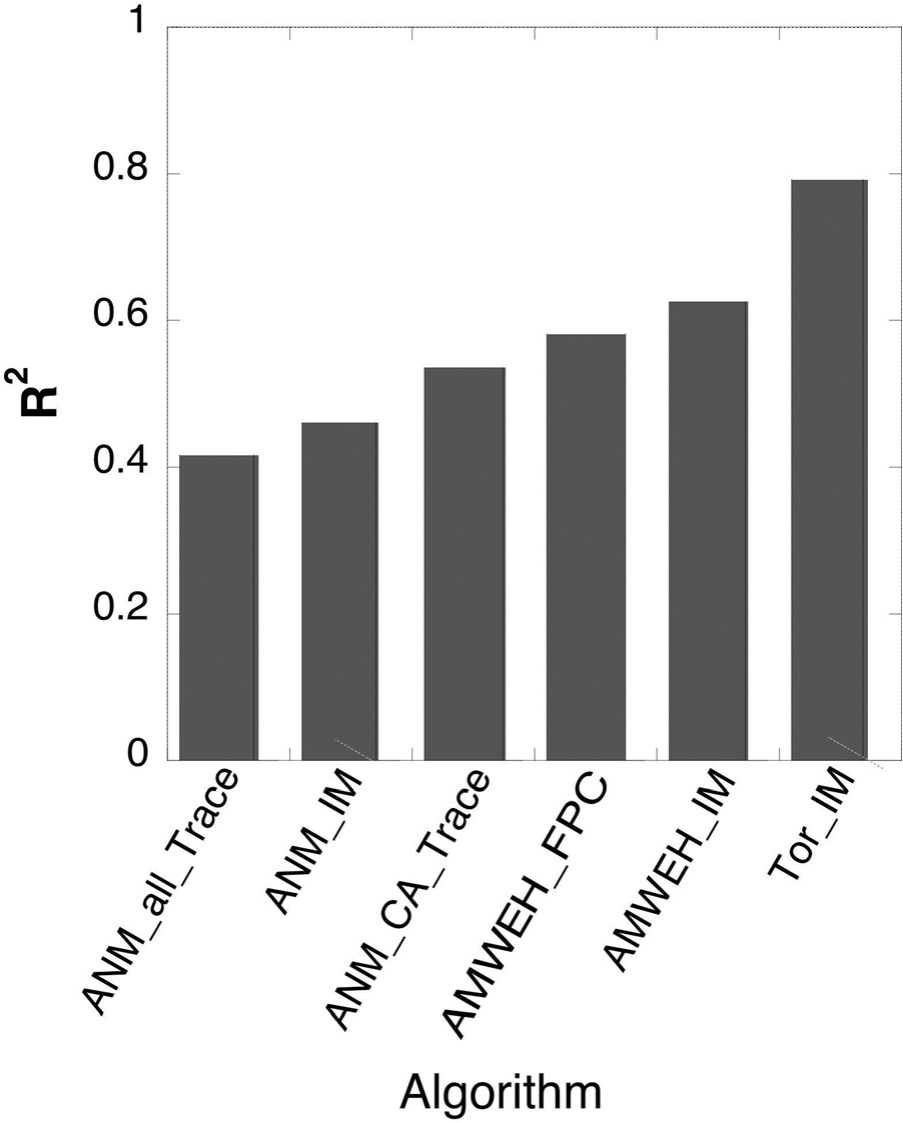
Enhanced agreement of PATH convergence parameters with experimental Δ*G_kcat_* values achieved by algorithmic changes discussed here and in Ref. 9. ANM, the original anisotropic network model used by Franklin *et al.*;^19^ AMWEH, the AMBER-based Mass-Weighted Empirical Hessian;^21^ Trace, the force constants estimated from the trace of the diagonalized Hessian matrix; FPC, the most significant eigenvalue of the diagonalized Hessian matrix; IM, the inverse mean algorithm described above.

Three points should be noted: (i) Use of both TOR_IM and AMWEH_IM values introduces two independent equations per variant and allows an assessment of statistical significance. The squared correlation coefficient is ∼0.9, 12 of 16 coefficients are statistically significant, with P-values for the estimates <0.005. (ii) As with the regressions of experimental Δ*G_kcat_* values against the mutant sites, regression coefficients emphasize the relative importance of higher-order interactions. (iii) Statistical significance cannot be assessed for similar histograms for the remaining PATH-derived parameters because the TOR_IM and AMWEH_IM values are uncorrelated owing to differences in the atomic models (all-atom, CA only) and hence support only one equation per variant. Their histograms (not shown) nevertheless differ distinctively from one another, arising from important contributions of opposite sign from different side chains and also emphasize the importance of high order interactions. As we could not demonstrate similar correlations using parameters derived from simpler PATH algorithms (i.e., those whose parameters were also less well correlated with experimental rate data (see Fig. 6), these observations suggest that, of the successive algorithms we tested, the revised torsional Hessian matrix used in the PATH algorithm is uniquely capable of detecting subtle structural changes and responding with appropriate stationary behavior.

Consideration of a large number of potential regression models between the diverse PATH convergence parameters and the experimental Δ*G_kcat_* values identified one that appears close to optimal in requiring only four adjustable coefficients in addition to the constant term. That model, represented in Fig. 5 and Table I, has a high *R*^2 ^= 0.81 and a highly significant F-ratio test (P < 0.0001), as well as highly significant Student t-test P-values (all <0.0001).

**TABLE I.** Regression model relating PATH convergence parameters to experimental $\Delta G_{kcat}$.

Term	Estimate	Std Error	t Ratio	Prob > |t|
Intercept	94.07	16.69	5.64	<0.0001
Δ*G*	0.16	0.03	5.43	<0.0001
$\left\langle U^{‡}\right\rangle$	−0.02	0.00	−5.28	<0.0001
$\left(-\right)\ln\left({\bar{t}}_{l}\right)$	17.47	3.22	5.43	<0.0001
$\Delta G\times \left(-\right)\ln\left({\bar{t}}_{l}\right)$	−1.48	0.26	−5.71	<0.0001

Innovations described in this paper—parameterizing torsional energy terms in the Hessian matrix and evaluating the trajectory in energy space instead of at different times—have emerged from a steady search for ways to improve the correlations between PATH parameters and the experimental kinetics data. It is worth illustrating these algorithmic enhancements by comparing *R*^2^ for the regression model in Table I with values obtained using the same regression model for the original and several intermediate algorithms (Fig. 6). The improvement evident in the histogram tells only part of the story, however. As the correlation improves, so do the Student t-values with corresponding reduction in their P-values. The statistical significance of the predictors and corresponding confidence in their values also improve markedly. For reference, the original ANM Hessian used in Ref. 9 (far left in Fig. 6) did not produce any significant t-test probabilities using the coefficients in Table I (1 > P > 0.4). Using the MWHH_IM algorithm improved *R*^2^ to 0.63 with (0.06 > P > 0.005). Adding the torsional potentials improved *R*^2^ to 0.81 with (P < 0.0001 for all values). Thus, improved agreement between predicted and experimental Δ*G_kcat_* values is associated with increased statistical confidence in the parameters of the regression model.

## CONCLUSION

**FIG. 7.**
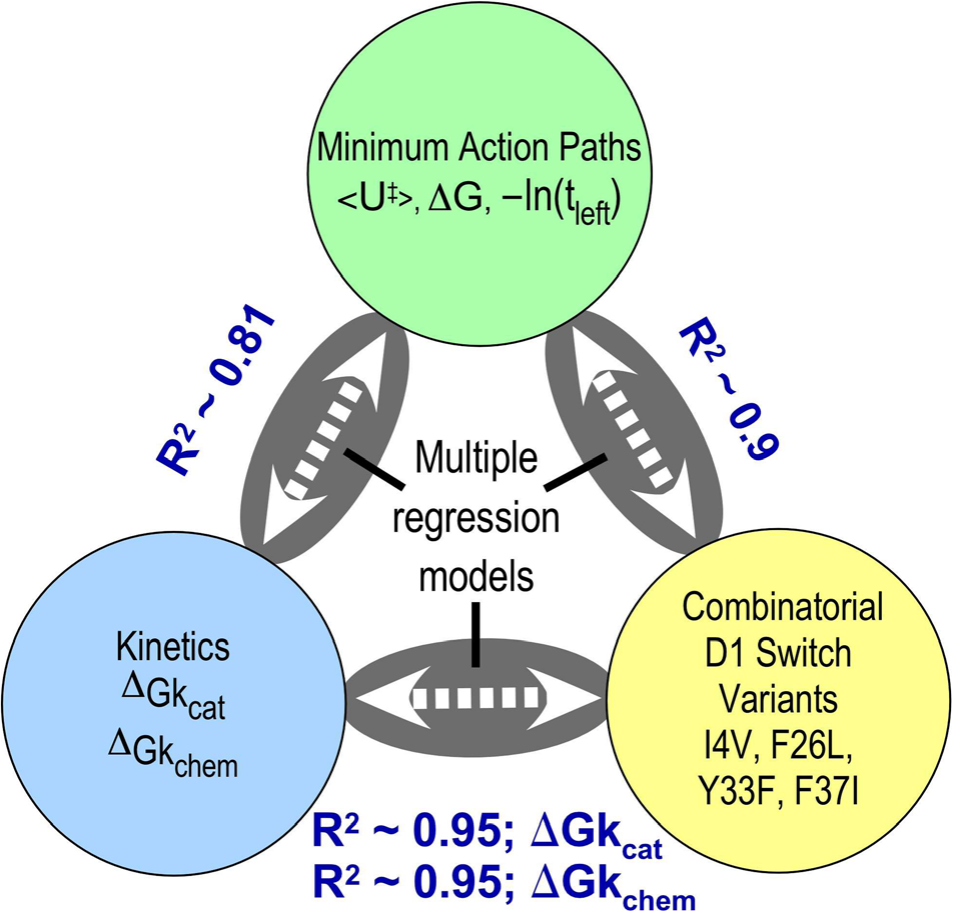
Interrelated statistical correlations that validate the enhanced algorithm provide a way to attribute cause and effect to physical properties of mutated side chains in combinatorial sets of variant proteins by establishing verifiable computational models of the trajectories and transition state structures of a coherent set of combinatorial mutants.^8^

The enhancements to the PATH algorithm described here increase only slightly the time taken to compute conformational change trajectories for TrpRS monomer (328 amino acids) (about 2%). The convergence parameters begin to be verifiable by comparison with experimental data. The correlations in Figs. 4 and 5 are compared in Fig. 7 with those of Ref. 42. Correlations along each edge of the triangle relate structural locations, enzyme kinetics data, and computational parameters to each other. The correlations between structure, experiment, and trajectory are quite significant, both in the fraction of the dependent variable variance that can be attributed to the independent variables and the statistical reliability of the coefficients. The correlations connecting the PATH parameters to the structural and experimental data are especially important to this diagram as they provide an independent link connecting the structural changes in the variant proteins to their impact on the experimental Δ*G_kcat_* values. Moreover, these dual correlations furnish a novel and comprehensive validation that simulated trajectories can help uncover relevant structural details about transient species that assist the interpretation of experimental data on protein structure and function.

We also note that this example is perhaps also unique in that for the first time, simulations can be used in a “high throughput” mode to compare meaningful computational parameters with experimental and structural data for ensembles of interrelated structures. This work demonstrates that computational analysis can complement experimental analysis of combinatorial mutants in a comparable time frame to enhance the value of higher-order combinatorial mutagenesis in identifying and measuring important free energy coupling between distant locations in conformationally dynamic proteins. The extensive and interconnected coupling patterns in Fig. 6 should ultimately be even more informative as we begin to be able to interpret functional coupling at high (i.e., inter-residue) resolution.

Finally, we should mention ways in which the PATH algorithm may be further improved. The transition state configurations we have encountered all bring aromatic side chains into conflict. This conflict is apparent from the fact that the ring structures actually shrink in structures output by PATH close to the transition states. Building the torsional angle restraints into the Hessian reduces this pathology to some extent. However, parameterizing the contributions to the Hessian that would restrain the planarity and size of the ring structures would address that problem more directly, and judging from the improvement evident in Fig. 6 we would expect still further improvement upon implementing those restraints.

## APPENDIX A: CONSTRUCTION OF THE TORSIONAL HESSIAN MATRIX

The interaction between atoms in PATH^9,19^ is represented by a Hessian Matrix, *H_ANM_*, which is a 3*N* × 3*N* matrix in which each element is a force constant for the interactions between a pair of atoms. These force constants are also the second derivative of the potential energy function. In the case of ANM, the elements are

$$h_{ij}=\frac{\partial^{2}V_{ANM}}{\partial x_{i}\partial x_{j}},$$ (A1)

where *x_i_* and *x_j_* are the Cartesian coordinates of the atoms *i* and *j* in the macromolecule.

Additional interaction terms can be added to the potential energy function by defining a new Hessian matrix and the overall interaction matrix is generated by adding the new Hessian matrix to the old one. Hence, we defined a new Hessian matrix for the torsional interaction, the torsional Hessian, *H_TOR_*, which can be constructed as follows.

Torsional potential energy is the interaction between atoms that are a part of a dihedral angle, defined between four atoms that are covalently bound in sequence. Torsional potential energy is mathematically expressed as

$$V_{TOR}=k_{\phi }\left(1-\text{cos}\;\left(n\left(\phi -\phi_{0}\right)\right)\right),$$ (A2)

where $k_{\phi }$ is the torsional force constant and $\phi_{0}$ is the dihedral angle at equilibrium.

Based on the assumption in Ref. 32, we consider $k_{\phi }=1$ and *n* = 1,

$$V_{TOR}=\left(1-\text{cos}\;\left(\phi -\phi_{0}\right)\right).$$ (A3)

Since the displacement from the equilibrium position is small in the case of macromolecular conformational changes, using Taylor's expansion, for small deviations from equilibrium, (A3) is written as

$$V_{TOR}=\frac{{\left(\phi -\phi_{0}\right)}^{2}}{2}.$$ (A4)

Note that this approximation resembles the Hooke's law potential energy of the ANM but is a higher-order interaction term because it implicates four, rather than two interacting atoms. Since the Hessian elements are second derivatives of the potential energy function, we have to calculate $\frac{\partial^{2}V_{TOR}}{\partial x_{i}\partial x_{j}}$, which can be written as

$$\frac{\partial^{2}V_{TOR}}{\partial x_{i}\partial x_{j}}=\frac{\partial \phi }{\partial x_{i}}\frac{\partial \phi }{\partial x_{j}}.$$ (A5)

Consider the four atoms that form the dihedral angle to be $a=\left(x_{1},y_{1},z_{1}\right),\;b=\left(x_{2},y_{2},z_{2}\right),\;c=\left(x_{3},y_{3},z_{3}\right)$, and $d=\left(x_{4},y_{4},z_{4}\right)$. Then, the difference between vectors can be computed as $v_{1}=\left(b-a\right)\Rightarrow \left(x_{2}-x_{1},y_{2}-y_{1},z_{2}-z_{1}\right),\;v_{2}=\left(c-b\right)\Rightarrow \left(x_{3}-x_{2},y_{3}-y_{2},z_{3}-z_{2}\right)$, and $v_{3}=\left(d-c\right)\Rightarrow \left(x_{4}-x_{3},y_{4}-y_{3},z_{4}-z_{3}\right)$. If the normal vectors to the planes containing the vectors *v*_1_, *v*_2_, and *v*_3_ are *w*_1_ and *w*_2_, where *w*_1_ = *v*_1_ × *v*_2_ and *w*_2_ = *v*_2_ × *v*_3_, then the dihedral angle ϕ is

$$\phi ={\text{cos}}^{-1}\left(\frac{w_{1}w_{2}}{\left|w_{1}||w_{2}\right|}\right).$$ (A6)

Assuming $R=\frac{w_{1}w_{2}}{\left|w_{1}||w_{2}\right|}$,

$$\frac{\partial \phi }{\partial X}=\frac{-1}{\sqrt{1-R^{2}}}\frac{\partial R}{\partial X},$$ (A7)

where *X* is the Cartesian coordinate.

Then, $\frac{\partial R}{\partial X}$ can be calculated as

$$\frac{\partial R}{\partial X}=\frac{1}{{\left(\left|w_{1}||w_{2}\right|\right)}^{2}}\left[|w_{1}||w_{2}|\left(\frac{\partial w_{1}}{\partial X}w_{2}+\frac{\partial w_{2}}{\partial X}w_{1}\right)-\left(w_{1}w_{2}\right)\left(\frac{\partial |w_{1}|}{\partial X}|w_{2}|+\frac{\partial |w_{2}|}{\partial X}|w_{1}|\right)\right].$$ (A8)

Since four atoms define each dihedral angle, for every group of four atoms a 12 × 12 block Hessian matrix, *h_ijkl_*, can be built using Equation (A5) for each pair of atoms in this group. Then, the 12 × 12 block matrix can be assembled into the full torsional Hessian, *H_TOR_*, using the procedure for assembling the ANM Hessian, *H_ANM_*, outlined in Ref. 9.

## APPENDIX B: CALCULATION OF TRAJECTORIES AS A FUNCTION OF ENERGY

To calculate the time taken to reach the transition state, we consider a single potential energy well where the system begins at the equilibrium state and at time $\bar{t}$ reaches the transition state. This can be mathematically written as $x_{2}=\bar{x},\;x_{1}=a,\;t_{1}=0$ and $t_{2}=\bar{t}$.

Then, the trajectory equation (3) can be rewritten as

$$x\left(t\right)=a+\frac{1}{\sinh\left(\Gamma \bar{t}\right)}\left(\left(\bar{x}-a\right)\sinh\left(\Gamma t\right)\right).$$ (B1)

Since the system has to be given enough time to converge, $\bar{t}\to \infty$,

$$x\left(t\right)-a=\left(\bar{x}-a\right)e^{\Gamma \left(t-\bar{t}\right)}.$$ (B2)

On squaring both sides

$$\frac{{\left(x\left(t\right)-a\right)}^{2}}{{\left(\bar{x}-a\right)}^{2}}=e^{2\Gamma \left(t-\bar{t}\right)}.$$ (B3)

In the case of ANM, since $U\left(x\right)=\frac{k{\left(x-a\right)}^{2}}{2}$, we can rewrite the above equation as

$$\frac{U\left(x\right)}{U\left(\bar{x}\right)}=e^{2\Gamma \left(t-\bar{t}\right)}.$$ (B4)

From (B4), *t* can be expressed as

$$t=\bar{t}+\frac{1}{2\Gamma }\ln\left(\frac{U\left(x\right)}{U\left(\bar{x}\right)}\right).$$ (B5)

As mentioned in Ref. 9 the condition for convergence is not just $\bar{t}\to \infty$ but $\Gamma \bar{t}\to \infty$. Hence $\bar{t}=\frac{X}{\Gamma }$, where *X* is large. Substituting this result in (B5), we get

$$t=\frac{X}{\Gamma }+\frac{1}{2\Gamma }\ln\left(\frac{U\left(x\right)}{U\left(\bar{x}\right)}\right),$$ (B6)

which can also be written as

$$t=\frac{1}{\bar{k}}\left[X+\frac{1}{2}\ln\left(\frac{U\left(x\right)}{U\left(\bar{x}\right)}\right)\right].$$ (B7)
